# Contrasting influences of aerosols on cloud properties during deficient and abundant monsoon years

**DOI:** 10.1038/srep44996

**Published:** 2017-03-24

**Authors:** Nitin Patil, Prashant Dave, Chandra Venkataraman

**Affiliations:** 1 Interdiciplinary program in Climate Studies, Indian Institute of Technology Bombay, Powai, Mumbai, India; 2 Department of Chemical Engineering, Indian Institute of Technology Bombay, Powai, Mumbai, India

## Abstract

Direct aerosol radiative forcing facilitates the onset of Indian monsoon rainfall, based on synoptic scale fast responses acting over timescales of days to a month. Here, we examine relationships between aerosols and coincident clouds over the Indian subcontinent, using observational data from 2000 to 2009, from the core monsoon region. Season mean and daily timescales were considered. The correlation analyses of cloud properties with aerosol optical depth revealed that deficient monsoon years were characterized by more frequent and larger decreases in cloud drop size and ice water path, but increases in cloud top pressure, with increases in aerosol abundance. The opposite was observed during abundant monsoon years. The correlations of greater aerosol abundance, with smaller cloud drop size, lower evidence of ice processes and shallower cloud height, during deficient rainfall years, imply cloud inhibition; while those with larger cloud drop size, greater ice processes and a greater cloud vertical extent, during abundant rainfall years, suggest cloud invigoration. The study establishes that continental aerosols over India alter cloud properties in diametrically opposite ways during contrasting monsoon years. The mechanisms underlying these effects need further analysis.

The Indian monsoon is influenced by multiple complex factors, from local physical processes to large-scale forcing. The role of aerosols has received recent attention^1^,^2^,^3^,^4^,^5^,^6^,^7^,^8^,^9^,^10^. Many studies have focused on monsoon rainfall changes, which are mediated by slow changes in sea surface temperatures; when changes in the sea surface temperatures degrade the north–south temperature gradient in the northern Indian Ocean, circulation changes occur that are correlated with reduced monsoon rainfall^1^,^5^. More recently, rapid changes in radiative forcing, because of both anthropogenic and natural aerosols^6^,^7^,^8^,^9^,^10^,^11^, have been linked to increases in northward moisture transport and, consequently, increases in rainfall, on daily and monthly timescales. Over continental areas of north India, changes in aerosols were linked to asymmetric changes in precipitation, with increases west, and decreases east, of 80°E^8^. Some studies have identified the influences of spatially separated aerosols (dust and black carbon over Himalaya; dust outbreak over Africa) on observed increases of net diabatic heating rates in the middle to upper troposphere or a strengthened northward pressure gradient over the Arabian Sea, with consequent increases in synoptic scale moisture convergence over India.

Significant aerosol concentrations over the Indian subcontinent occur during the summer monsoon season; the aerosol levels correlate with cloud properties, as illustrated for the monsoon month of July^12^ and for the deficient monsoon year of 2009^13^. Different mechanisms by which aerosols mediate cloud and rainfall development have been proposed. Meteorological covariance can obscure observational evidence of the aerosol modification of clouds. However, recent observational studies have attempted to control for meteorological effects through the classification of clouds into regimes. Absorbing aerosols could lead to stabilization of the near-surface atmosphere, leading to positive feedback that reduces cloudiness^14^. Observational evidence from the Amazon biomass burning season supports the theory that black carbon aerosols inhibit warm cloud development^15^. Absorptive dust aerosol outbreaks over the Taklimakan desert^16^ and East Asia’s arid regions^17^, have been linked to large atmospheric warming effects and to significant reduction in the liquid and ice water path in dust-contaminated clouds^18^. In contrast, an increase in the availability of cloud condensation nuclei at the cloud base could enhance cloud “invigoration”, and increase rainfall intensity^19^,^20^. Observations support aerosol-mediated increases in the transition from stratocumulus to convective cloud regimes^21^ and rainfall intensity^22^. However, to the best of our knowledge, the modulation of monsoon clouds by both spatially and temporally coincident aerosols has received little attention.

This study focussed on three of six homogeneous monsoon rainfall regions identified on the basis of similarity in rainfall characteristics and association of sub-divisional monsoonal rainfall with regional/global circulation parameters^23^. The three selected regions, together account for over 85% of annual summer monsoon rainfall and constitute the “core monsoon zone” over the Indian subcontinent^24^,^25^. To investigate the aerosol modulation of clouds and rainfall during deficient and abundant monsoon years, we used observational data from June to September (JJAS), from 2000 to 2009. These data were coincident in space and time and include gridded monsoon rainfall^26^,^27^, aerosol optical depth (AOD) and cloud properties, sourced from the Moderate Resolution Imaging Spectroradiometer (MODIS) Terra and Aqua Level 3 satellites.

A seasonal normalized precipitation anomaly was used to identify deficient and abundant rainfall years in each region. The season mean variables in abundant and deficient rainfall years, respectively, were aggregated at a pixel level, to analyse the anomalies in coincident aerosol abundance and cloud properties, during the summer monsoon months of 2000–2009. The specific issue addressed in this work relates to whether the nature of aerosol modulation of cloud properties remain the same under distinct conditions encountered in different monsoon years, i.e. in deficient versus abundant monsoon years, do aerosols affect clouds in similar or dissimilar ways.

## Results

### Season mean rainfall, aerosol and cloud properties

**Figure 1:**
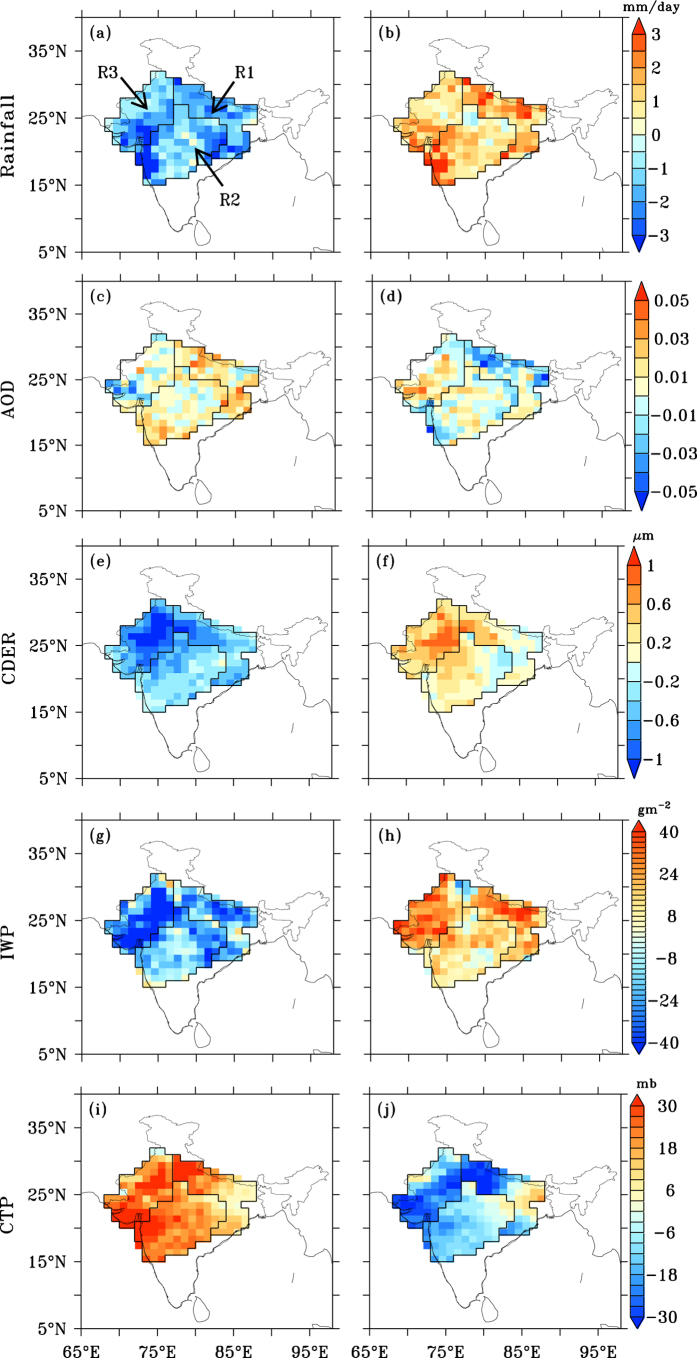
Spatial distribution of region-specific June–September (JJAS) anomalies during deficient (first column) and abundant (second column) rainfall years. (**a**,**b**) rainfall, (**c**,**d**) aerosol optical depth (AOD), (**e**,**f**) cloud drop effective radius (CDER, μm), (**g**,**h**) ice water path (IWP, gm^−2^) and (**i**,**j**) cloud top pressure (CTP, mb). Figure was created using FERRET v7.0 (http://www.ferret.noaa.gov/Ferret/).

The deficient and abundant rainfall years differed in the three regions studied (Supplementary Fig. S1). The combined rainfall anomalies, calculated at pixel level, for the “deficient” and “abundant” rainfall years in each region, ranged from −3 to 0 and 0 to +3 mm day^−1^, respectively, for over 95% of the pixels (Fig. 1a,b). Deficient rainfall years were characterized by a greater number of “break periods” than abundant rainfall years (Table S1); a break period was defined as three or more consecutive days with the normalized rainfall anomaly below −1 (ref. 25). During the deficient rainfall years, there were eight, eight and two break periods, respectively, in R1, R2 and R3; during abundant rainfall years there were one, four and zero break periods, respectively. The numbers of days that fell within the break periods were significantly larger during deficient years (35, 42 and 8 days, respectively, in R1, R2 and R3) than in abundant years (3, 21 and 0 days, respectively) (Table S1).

An understanding of the aggregated aerosol and cloud properties during deficient and abundant rainfall years would allow an analysis of how the aerosols mediate the cloud properties. Aerosol and cloud cannot be observed simultaneously at the scale of Level-1 MODIS retrievals, wherein only pixels identified as cloud-free are used for making Level-2 aerosol retrievals. However, for the Level-3 product (at 1° × 1°), both cloud-free aerosol retrievals and cloud retrievals are averaged, from the respective Level-2 datasets^28^, leading thereby to presence of both aerosol and cloud retrievals in the same Level-3 pixel. In the present dataset, 20–50% (70–190 data pairs) of daily retrievals at Level 3, contained both AOD and CDER data (Supplementary Fig. S2). We follow previous studies using the MODIS Level 3 product^12^,^13^,^22^ to investigate aerosol-cloud interactions. Aerosol build-up has been observed, even during the JJAS monsoon months; anthropogenic emissions and dust were seen to increase columnar aerosol abundance during rainfall break periods and sometimes even during active surface rainfall periods^29^, in the case of elevated dust plumes. During deficient monsoon rainfall years, largely positive anomalies in AOD were found (83% of pixels) while in abundant rainfall years these were largely negative (69% of pixels) (Fig. 1c,d). Contrasting AOD anomalies were also seen in the absolute AOD data and were larger in deficient than in abundant rainfall years for all regions, with high statistical significance (P < 0.10) (Supplementary Fig. S3a). Examination of the aerosol index, or the aerosol absorbing index (AAI), showed that absorbing aerosols had a similar behaviour to AOD (Supplementary Fig. S4a–c). This implies that the abundances of absorbing aerosols (dust and possibly black carbon), and those of the total aerosols, were larger in deficient monsoon years.

Key cloud properties, coincident with the aerosols detected, were significantly different between deficient and abundant rainfall years. Mean cloud drop size, measured through the cloud drop effective radius (CDER), had almost exclusively negative anomalies in all regions during the deficient rainfall years (Fig. 1e; 99% pixels) and positive anomalies during the abundant rainfall years (Fig. 1f; 84% pixels). The contrasts in the CDER anomalies were consistent with those of the CDER season mean for each region, being lower in the deficient, compared with the abundant rainfall years (Supplementary Fig. S3b) (P < 0.10). It is generally accepted that a critical cloud drop radius is necessary for settling and initiating auto-conversion processes; these processes mainly result from drop sweep-out by falling raindrops, leading to drop growth and the onset of precipitation. Mean cloud drop size (Supplementary Fig. S3b) observed during deficient rainfall years, particularly in R3, could inhibit rainfall development. These findings are consistent with more frequent observations of smaller cloud drop sizes in the month of July during deficient rainfall years^12^. In addition to the microphysical effects, the greater abundances of absorbing aerosols, discussed above, could potentially exert a radiative effect, through stabilization of the near-surface atmosphere^30^ and lead to the inhibition of vertical moisture transport. Season mean lower tropospheric stability (Supplementary Fig. S5) showed a positive anomaly in deficient monsoon years, in contrast to a negative or negligibly positive anomaly in abundant monsoon years, over almost all parts of the three selected regions, which merits further investigation.

Analyses of other cloud properties revealed that the deficient monsoon years were characterized by largely negative anomalies of the ice water path (IWP; 91% of pixels), but positive anomalies (98%) in cloud top pressure (CTP) (Fig. 1g and i). Abundant monsoon years were characterized by positive anomalies in IWP (93%), but negative anomalies in CTP (86%) (Fig. 1h and j). Region mean values of IWP (Supplementary Fig. S3c) were lower (higher), but those of CTP were higher (lower) in all regions during deficient (abundant) rainfall years (P < 0.10). Similar anomalies were observed in cloud liquid water path (LWP; data not shown). At the mean cloud top height during abundant monsoon years (with a CTP of 410–580 mb) temperatures reached below freezing point (257–318 K), while during deficient rainfall years (CTP of 460–650 mb) the temperatures were largely above freezing (312–317 K). These results are consistent with observations of larger cloud vertical extents, and greater liquid and ice water contents during active monsoon spells^31^, which occur more frequently in abundant rainfall years.

The relationships between aerosol abundance and different cloud properties were first examined through correlation analyses of the season mean values at pixel level (Table 1); this was done within each homogeneous rainfall region, to limit the spatial extent and attempt to avoid spurious correlations^32^ from climatological gradients of aerosol and cloud properties, which may occur over larger spatial scales. During the season in deficient monsoon years, negative correlations were found between AOD and cloud drop size, in two of the three regions (Table 1). This indicates that higher aerosol abundances were coincident with lower cloud drop sizes. In contrast, a decrease in cloud drop size was not found with increasing AOD in abundant monsoon years, in two of the three regions. Season mean column water vapor availability showed a negative anomaly in deficient, but a positive anomaly in abundant monsoon years (Supplementary Fig. S6), indicating a combined effect of aerosols and water vapour limited regime on reduced cloud drop sizes in deficient rainfall years.

Correlation analyses of the season mean AODs with CTP and IWP showed differing behaviours among the regions (Table 1). In R3, northwest India, AOD was correlated positively with CTP, during both deficient and abundant rainfall years. In addition, increases in AOD were correlated with decreases in IWP, or reduced ice processes, during deficient monsoon years, but there were no significant correlations during abundant rainfall years. This suggests that greater aerosol abundances were coincident with shallower clouds and decreased ice processes, during deficient rainfall years. These patterns differed from those observed in R1 and R2. R1 and R2 were identified in previous studies^10^ as part of the core monsoon zone. In R1 and R2, the aerosol abundances were correlated with shallower clouds, but increased ice processes, during deficient monsoon years. During abundant rainfall years, there were no significant relationships between aerosol abundance and either cloud vertical extent or ice processes. The clouds were shallowest in R3 compared with the other regions (Supplementary Fig. S3d; P < 0.10). As the interactions between aerosols and clouds occur on short timescales, the aerosol–cloud relationships based on the season mean, as discussed above, were further evaluated with analyses at daily timescales, below.

### Daily mean aerosol and cloud properties

**Figure 2:**
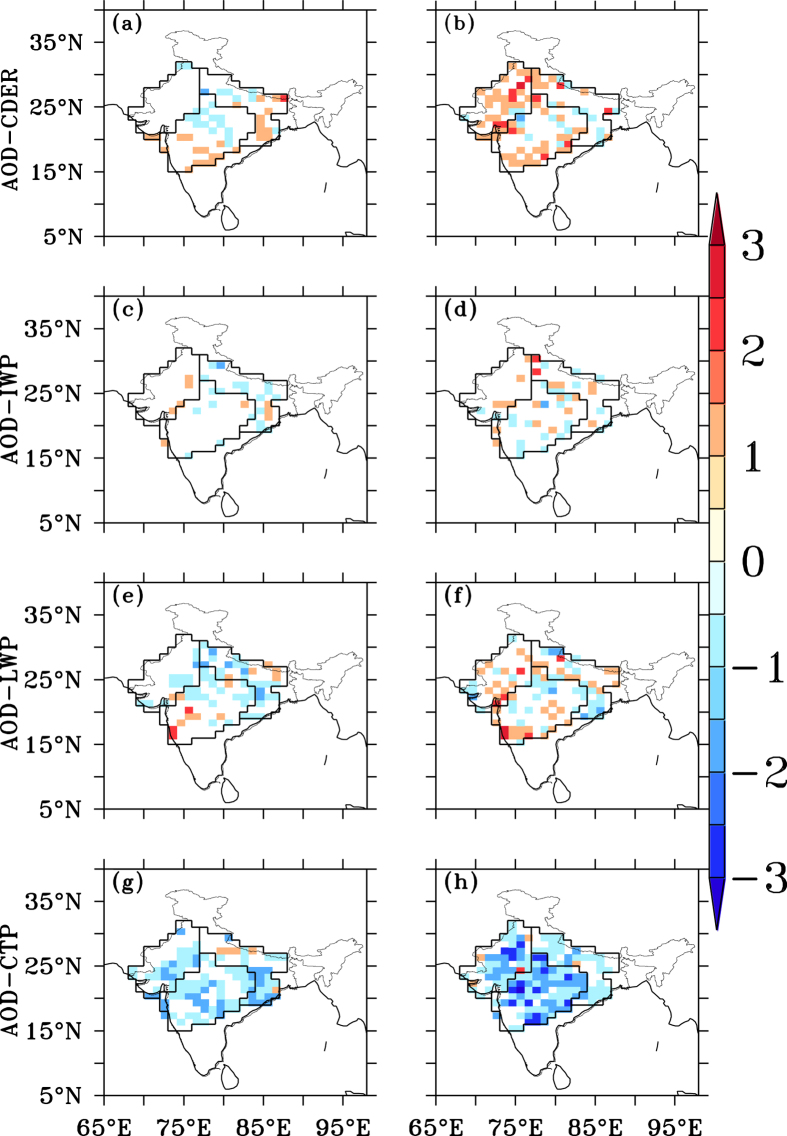
Temporal correlation cumulative frequency analyses during deficient (first column) and abundant (second column) rainfall years. (**a**,**b**) AOD and CDER; (**c**,**d**) AOD and IWP, where cloud top temperature (CTT) <0°; (**e**,**f**) AOD and liquid water path (LWP); and (**g**,**h**) AOD and CTP. Correlations are significant at P < 0.1. Figure was created using R statistical tool v3.3.1 (https://www.r-project.org/) and FERRET v7.0 (http://www.ferret.noaa.gov/Ferret/).

Temporal correlation analyses between aerosols and cloud properties, of the daily mean values at pixel level, were carried out for each year, from 2000 to 2009 (Fig. 2). In each region (R1, pixels 59; R2, pixels 87; and R3, pixels 63) for every year, the correlations between AOD and the cloud properties (CDER, IWP, LWP and CTP) were calculated using the absolute values of each parameter. The statistical significance of correlation coefficient was tested at α = 0.10. At a given pixel, to identify the cumulative frequency of occurrence, a correlation greater than zero was assigned as + 1 and a correlation less than zero was assigned −1. The cumulative sums of these values were calculated for deficient and abundant years separately; larger, positive values of cumulative frequency indicated the presence of more number of positive correlations and vice-versa.

**Figure 3:**
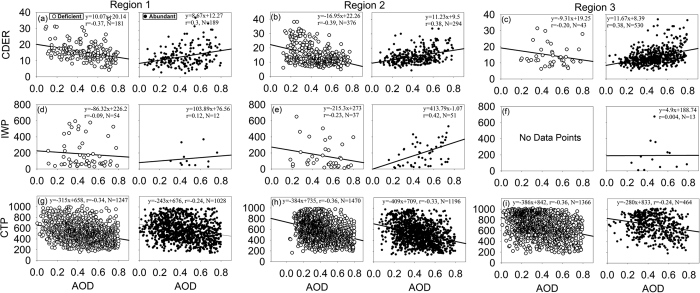
Scatter plot of statistically significant AOD–cloud property correlations at the pixel level, for R1, R2 and R3, during deficient and abundant rainfall years. (**a–c**) AOD and CDER (μm); (**d–f**) AOD and IWP (gm^−2^); and (**g**–**i**) AOD and CTP (mb). Figure was created using SigmaPlot v11 (https://systatsoftware.com/).

During deficient rainfall years, negative correlations occurred between AOD and CDER more often than positive correlations, while the opposite pattern was observed during abundant rainfall years (Fig. 2a, b). This contrast was observed most prominently in R2 and R3. At the daily timescale, greater aerosol abundances were thus coincident with decreased (increased) cloud drop sizes in deficient (abundant) rainfall years. The negative correlations between AOD and CDER during deficient periods, and positive correlations during abundant periods, were statistically significant in all three regions (Fig. 3a–c), based on the composited analyses. Thus, an opposing influence of aerosol abundance on cloud drop size is evident; higher levels of aerosols lead to smaller cloud drop sizes in deficient, and larger size in abundant, rainfall years.

The relationships between daily-mean AOD and IWP were examined by analysing the pixels with cloud top temperatures (CTTs) less than 0 °C (Fig. 2c,d). The behaviour was different among regions. In R1 and R2 negative correlations between AOD and IWP occurred more frequently during deficient rainfall years, while positive correlations were more frequent during abundant rainfall years (Fig. 3d–f). In R3, during deficient years there were not enough data points to analyse the relationship statistically, but no significant correlations were seen during the abundant rainfall years. Thus aerosols were related with a decrease in ice processes during deficient, but an increase in ice processes during abundant, rainfall years.

The relationships between AOD and LWP, were positive in both abundant and deficient rainfall years (Fig. 2e,f); positive correlations occurred more frequently during the abundant rainfall years. This implies that increases in aerosol abundance, with possibly greater availability of CCN, particularly sea salt aerosols entrained in the strong westerly monsoon flows^33^, were correlated with greater amounts of liquid water in the clouds.

The AOD displayed negative anomalies in the abundant rainfall years, consistent with the rainout of aerosols. Within this depleted aerosol field, the correlations between AOD and CTP (Fig. 2g,h) were more frequently negative during abundant, than deficient, rainfall years. This indicates that with an increase in aerosol abundance, lower CTP, or higher cloud heights, occurred during the abundant monsoon years. However, the correlations between CTP and AOD (Fig. 3g–i) were not significantly different between the abundant and deficient rainfall years; this indicates that the magnitude of this effect did not change much between abundant and deficient rainfall years. We note that fewer daily data were available during the abundant monsoon years, because of missing AOD values in the satellite retrieval data.

## Discussion

We used independent, but temporally and spatially coincident, data sets of rainfall data, and aerosol and cloud property data. We established contrasting correlations between aerosol abundance and cloud properties on season mean and daily mean timescales, during deficient and abundant monsoon years. The effects of aerosols act largely through microscale to mesoscale alterations of cloud microphysics and macrophysics, possibly through the alteration of lower atmospheric stability on sub-seasonal timescales. The contrasts in the correlations during deficient and abundant rainfall years, of AOD with CDER, IWP and CTP, indicate that the abundance of aerosols correlated with smaller (larger) cloud drop sizes during deficient (abundant) rainfall years. Increases in aerosol abundance were correlated with a greater persistence of lower ice processes and shallower cloud heights during deficient rainfall years, but taller clouds and, evidence of, greater ice processes, during abundant rainfall years.

Deficient monsoon years are characterized by lower levels of moisture convergence, vertical velocity and available column water vapour^24^,^34^. However, there is significant evidence of physical mechanisms that could explain the observed correlations between aerosol and cloud properties, while exercising caution in any interpretation. Satellite observations support the instantaneous brightening of clouds with increases in aerosol abundance, through a decrease in CDER^35^ and a corresponding increase in cloud drop number concentration^36^. In addition to the microphysical effect, the presence of absorbing aerosols could potentially exert a radiative effect, through stabilization of the near-surface atmosphere because of aerosol absorption^14^, which inhibits moisture transport and cloud development. Negative correlations of AOD-CDER, along with a positive anomaly in season mean lower tropospheric stability, found here in deficient monsoon years, are consistent with mechanisms suggested in previous studies.

Alternately, the aerosol–cloud invigoration mechanism^19^,^37^, postulates that a larger abundance of aerosols at the base of warm convective clouds leads to greater vertical cloud extent, and increased invigoration, which is supported by observations^22^,^38^. An independent effect of aerosols on convective cloud invigoration over the Atlantic, was suggested through differences in correlations of meteorological parameters with observed aerosol optical depth from those with convective cloud properties, both in terms of the meteorological variables concerned and the correlation sign^30^. Aerosol loading was linked to invigoration of convective clouds leading to increased cloud macroscopic properties like cloud-top heights, thicknesses, and the expansion of anvil cloud fractions, in unstable, moist atmospheres in the US Great Southern Plains^39^. Positive relationships found between cloud drop effective radius and aerosol optical depth or aerosol total concentration, in the Gulf of Mexico and South China Sea, were reproduced in model calculations upon the introduction of giant CCNs^40^. Over India, observations reveal a higher availability of sea-salt CCN in abundant monsoon years from entrainment in strong on-shore westerly flows^33^.

Negative AOD–CTP correlations and positive AOD-CDER correlations, seen in this study, occurred more frequently in abundant monsoon years, along with more frequent, positive correlations between AOD and CF (Supplementary Fig. S7a,b). Negative AOD–CTP correlations observed in previous studies, were largely explained by a positive relationship between AOD and cloud fraction (CF)^22^. While AOD–CF relationships are sometimes influenced by a high-bias in aerosol retrieval, from cloud contamination or meteorological effects like aerosol humidification, these relationships have been observed using different measurement systems^22^; this suggests that they are not caused by measurement artefacts alone. The present findings imply that, when the overall greater convective activity and vertical moisture transport in abundant monsoon years is coincident with higher aerosol concentrations, deeper and taller convective clouds occur.

The main finding of the work is the opposing nature of aerosol modification of clouds in deficient versus abundant monsoon years. Overall, during deficient rainfall years, greater aerosol abundance was correlated with a smaller cloud drop size, the persistence of lower ice processes and shallower cloud heights, consistent with aerosol-mediated inhibition of cloud development. In contrast, during abundant rainfall years, aerosol abundance was correlated with a larger cloud drop size, taller clouds and greater ice processes; this implied that cloud invigoration was mediated by the coincidence of aerosols and stronger convective fields. The coincidence of aerosols and taller clouds suggests that vapour accretion during the longer duration of entrainment may explain, in part, the observed positive correlations between AOD and CDER during abundant monsoon years. Other studies have shown very important responses of the monsoon aerosol-induced changes that have acted through sea surface temperatures (linked to monsoon weakening)^1^,^5^,^41^ and through temperature and pressure gradients, which facilitate moisture convergence and rainfall onset^6^,^7^,^8^,^9^,^10^,^11^. However, the consistent correlations seen in this work, from the daily to season timescales, show that coincident continental aerosols alter cloud properties, in diametrically opposite ways, in contrasting monsoon years. A suggestive implication of the findings is that aerosols inhibit cloud development in deficient, but invigorate it in abundant, monsoon years. Further work is needed to carefully attribute the observed relationships to the causal factors that could affect the consequent rainfall development.

## Methods

### Aerosol and cloud properties: MODIS Terra and Aqua Level 3 Data

Satellite measurements of cloud properties, including microphysical, optical and thermodynamic properties were used. Data included CDER, LWP, IWP, CF, CWV, CTP and CTT. Aerosol properties, including AOD, were used as measures of the columnar abundance of total and absorbed aerosols, respectively. All variables, except AAI, were downloaded from the NASA GES DISC Giovanni online data system (http://gdata1.sci.gsfc.nasa.gov/daac-bin/G3/gui.cgi?instance_id=MODIS_DAILY_L3). The data from the MODIS remote sensors on board the earth observing system (EOS) terra and aqua satellites, level 3, were used at 1° × 1°, latitude × longitude, resolution^42^ for the months of JJAS, from 2000 to 2009. The MODIS instruments are flown on the Terra and Aqua satellites in sun-synchronous orbits, with equatorial crossing times of 1030 and 1330 LST, respectively. These instruments are of the same design, to reduce the error due to instrument differences. All the data products used from 2000 to 2002 were from the Terra satellite; data products from the Aqua satellite are available from July 2002 onwards. During 2003–2009, MODIS data products obtained from both the Terra and Aqua satellites were combined and utilized. Earlier studies^43^ found that there are smaller differences between land and ocean AOD data from MODIS, compared with ground-based aerosol robotic network (AERONET) sun/sky radiometer measurements^44^. Since the season is characterized by the prevalence of cloudy skies, AODs larger than 0.8 were excluded from the data sets. This avoided potentially large influences of satellite retrieval errors, such as cloud contamination, or domination by aerosol swelling, from the large relative humidity around clouds. Cloud properties from MODIS L3 were derived from MODIS Atmosphere L3 Gridded Product Algorithm^28^. Cloud droplet effective radius retrievals from MODIS are made from the 1.6, 2.1, and 3.7 μm bands, along with analyses and L3 aggregations that enable improved spectral retrieval intercomparisons and estimate quantitative pixel level uncertainty (https://modis-atmos.gsfc.nasa.gov/_docs/C6MOD06OPUserGuide.pdf). In marine stratocumulus clouds, MODIS retrieved cloud effective radius using the 2.1 μm wavelength channel overestimated *in situ* measurements on average by 13% (ref. 46). Passive remote sensed cloud products have been widely used in previous work^12^,^13^,^22^,^31^ investigating aerosol-cloud interactions. While data have recently become available, after 2006, from active sensors, like the Cloud-Aerosol Lidar with Orthogonal Polarization (CALIOP), a two-wavelength polarization lidar^47^, and a cloud profiling radar^31^, these datasets remain somewhat limited in terms of spatial and temporal coverage.

### Rainfall observations

Daily mean rainfall data (1° × 1°), provided by the Indian Meteorological Department (IMD) were used for the precipitation analysis from 2000 to 2009. The rainfall data set^27^ included rainfall data from 2140 stations. Deficient and abundant rainfall years in each region, were identified through the calculation of a seasonal normalized precipitation anomaly, for the monsoon months of JJAS, during the ten year period of 2000–2009.

### Aerosol absorbing index (AAI): TOMS and OMI

AAI data were from the Total Ozone Mapping Spectrometer (TOMS, 1° × 1.25°) for 2000–2004^48^ and the ozone monitoring instrument (OMI, 1° × 1°) for 2005–2009^49^, as per their availability. To calculate anomalies, for the 10-year period (2000–2009), in the abundant and deficient monsoon years we interpolated the OMI data set on the TOMS grid (i.e. 1° × 1.25°).

### Deficient and abundant rainfall years

In each region, deficient and abundant rainfall years, during the 10-year period (2000–2009), were identified through calculation of a seasonal normalized precipitation anomaly, for the monsoon months of JJAS (Supplementary Fig. S1). The normalized anomalies were calculated at the pixel level as the ratio between the difference of the annual mean from the 10-year mean, and the standard deviation of the 10-year mean values. A threshold anomaly value of ± 0.1 was used to define years as “abundant” (>0.1) and “deficient” (<−0.1) rainfall, consistent with the threshold used for the whole country (Ref: IMD Technical Circular No. 2/2007). Deficient rainfall years in R1 were 2002, 2004 and 2009; in R2 and R3 they were 2000, 2002 and 2009. Abundant rainfall years in R1 were 2003, 2007, 2008; in R2 they were 2003, 2005, 2006, and 2007; and in R3 they were 2003, 2006, 2007, and 2008 (Supplementary Fig. S1).

### Pixel-level temporal correlation analysis

In each region (R1, R2 and R3) the correlations between aerosol (AOD) and cloud properties (CDER, IWP, LWP, CTP and CF) were calculated for each year (individually), using absolute values of the AOD and cloud properties. Correlation coefficients were considered statistically significant (α) at 0.10. At a given pixel, correlations greater than zero were assigned as +1 and correlations less than zero were assigned as −1; to identify the cumulative frequency of occurrence, these values were aggregated across deficient and abundant years. Therefore, a positive value of cumulative frequency indicates the presence of more positive correlations, and vice-versa.

### Lower Tropospheric Stability (LTS)

Lower tropospheric stability (LTS = θ_700hPa_ − θ_1000hPa_; Kelvin), which is defined as the difference in potential temperature (θ) between the 700-hPa level and the surface^50^, anomalies were analysed using the ERA-Interim dataset produced by the European Centre for Medium-Range Weather Forecasts^51^.

## Additional Information

**How to cite this article:** Patil, N. *et al*. Contrasting influences of aerosols on cloud properties during deficient and abundant monsoon years. *Sci. Rep.* **7**, 44996; doi: 10.1038/srep44996 (2017).

**Publisher's note:** Springer Nature remains neutral with regard to jurisdictional claims in published maps and institutional affiliations.

## Supplementary Material

Supplementary Information

## Figures and Tables

**Table 1:** Correlations between season mean aerosol abundance and different cloud properties during deficient and abundant monsoon years in each region studied.

Region	Property	During deficient rainfall years				Region	Property	During abundant rainfall years			
		AOD	CDER	IWP	CTP			AOD	CDER	IWP	CTP
Region 1	AOD	1.00				Region 1	AOD	1.00			
	CDER	−0.06	1.00				CDER	−0.03	1.00		
	IWP	**0.33**	0.01	1.00			IWP	0.06	0.01	1.00	
	CTP	**0.23**	**−0.85**	**0.23**	1.00		CTP	0.09	**−0.81**	0.14	1.00
Region 2	AOD	1.00				Region 2	AOD	1.00			
	CDER	**−0.60**	1.00				CDER	**−0.13**	1.00		
	IWP	**0.26**	0.11	1.00			IWP	−0.1	−0.05	1.00	
	CTP	**0.44**	**−0.37**	0.03	1.00		CTP	0.04	**−0.52**	**0.18**	1.00
Region 3	AOD	1.00				Region 3	AOD	1.00			
	CDER	**−0.20**	1.00				CDER	−0.10	1.00		
	IWP	**−0.30**	**0.56**	1.00			IWP	0.08	**0.54**	1.00	
	CTP	**0.24**	**−0.56**	**−0.23**	1.00		CTP	**0.39**	**−0.65**	**−0.33**	1.00

Bold indicates a statistically significant correlation (P < 0.10).
