# Supplementary material for: Contrasting influences of aerosols on cloud properties during deficient and abundant monsoon years
Source: Sci Rep. 2017 Mar 24;7:44996. doi: 10.1038/srep44996 (PMC5364465; doi:10.1038/srep44996)
Supplement: Supplementary Information [file srep44996-s1.pdf]

Contrasting influences of aerosols on cloud properties during deficient and abundant monsoon years

Nitin Patil[1], Prashant Dave[1], Chandra Venkataraman[*,1,2]

[1]Interdiciplinary program in Climate Studies, Indian Institute of Technology Bombay, Powai, Mumbai, India
[2]Department of Chemical Engineering, Indian Institute of Technology Bombay, Powai, Mumbai, India

---

**Corresponding Author*:

Prof. Chandra Venkataraman
Professor of Chemical Engineering
Convener, Interdisciplinary Programme in Climate Studies
Indian Institute of Technology Bombay
Powai, Mumbai - 400 076 (INDIA)
E-mail: chandra@iitb.ac.in
Phone: +91-22 2576 7224
Fax: +91-22- 2572 6895 / 3480

List of figures and tables in Supplementary Information

Figure S1 | Season (JJAS) precipitation normalized anomalies over R1 (central northeast), R2 (west central) and R3 (northwest). Used to identify region-specific abundant and deficient rainfall years.

Figure S2 | Frequency count (as percent of total daily retrievals) of AOD and CDER pairs at each pixel during (a) deficient and (b) abundant rainfall years. Figure was created using R statistical tool v3.3.1 (https://www.r-project.org/) and FERRET v7.0 (http://www.ferret.noaa.gov/Ferret/).

Figure S3 | Box and Whisker plot of region-specific JJAS absolute values at the pixel level during deficient (grey) and abundant (white) rainfall years. (a) AOD, **(b)** CDER, **(c)** IWP and **(d)** CTP. Mean values during deficient and abundant rainfall years are significantly different at $P < 0.10$.

Figure S4 | Region-specific aerosol absorbing index JJAS anomalies during (a) deficient and (b) abundant rainfall years; and (c) Box and Whisker plot of the absolute values at each pixel. Figure was created using FERRET v7.0 (http://www.ferret.noaa.gov/Ferret/).

Figure S5 | Region-specific lower tropospheric stability (LTS) JJAS anomalies during (a) deficient and (b) abundant rainfall years. Figure was created using FERRET v7.0 (http://www.ferret.noaa.gov/Ferret/).

Figure S6 | Region-specific column water vapour JJAS anomalies during (a) deficient and (b) abundant rainfall years. Figure was created using FERRET v7.0 (http://www.ferret.noaa.gov/Ferret/).

Figure S7 | Temporal correlation cumulative frequency of AOD and CF during (a) deficient and (b) abundant rainfall years (significant at $P < 0.10$). Figure was created using R statistical tool v3.3.1 (https://www.r-project.org/) and FERRET v7.0 (http://www.ferret.noaa.gov/Ferret/).

Table S1. Number of break periods and days per break period during abundant and deficient rainfall years, based on the Indian Meteorological Department rainfall observations.

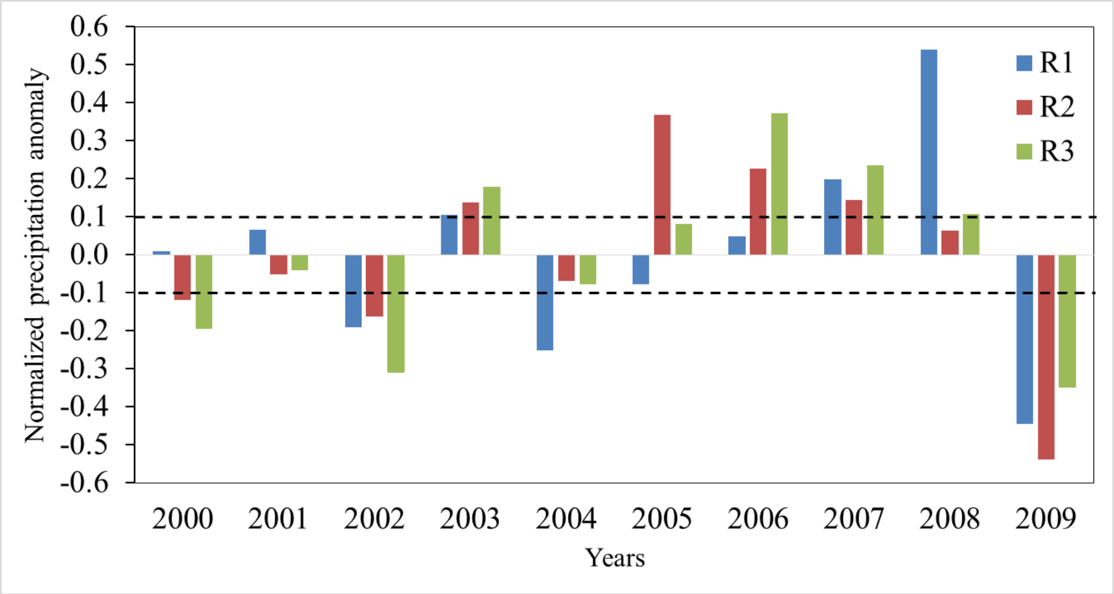

Figure S1 | Season (JJAS) precipitation normalized anomalies over R1 (central northeast), R2 (west central) and R3 (northwest). Used to identify region-specific abundant and deficient rainfall years.

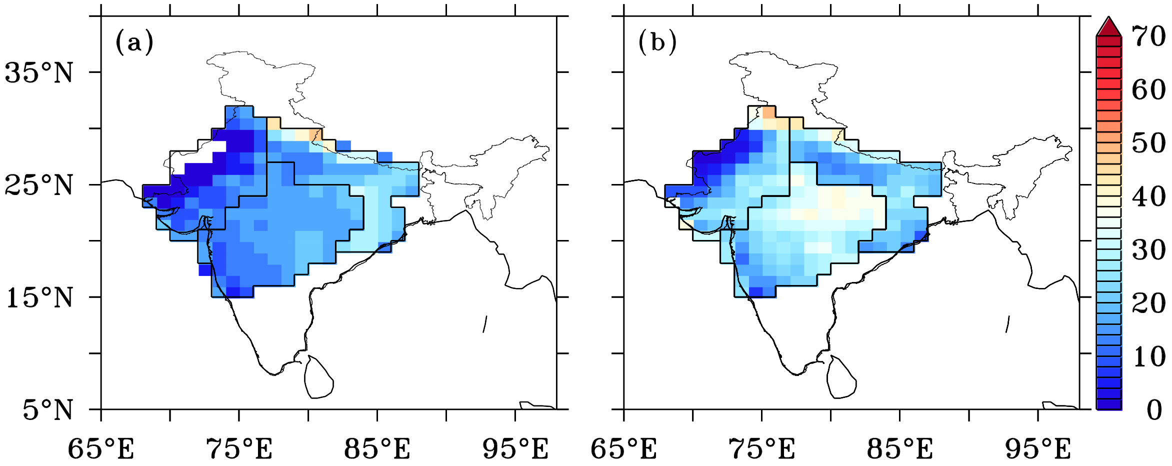

Figure S2 | Frequency count (as percent of total daily retrievals) of AOD and CDER pairs at each pixel during (a) deficient and (b) abundant rainfall years. Figure was created using R statistical tool v3.3.1 (https://www.r-project.org/) and FERRET v7.0 (http://www.ferret.noaa.gov/Ferret/).

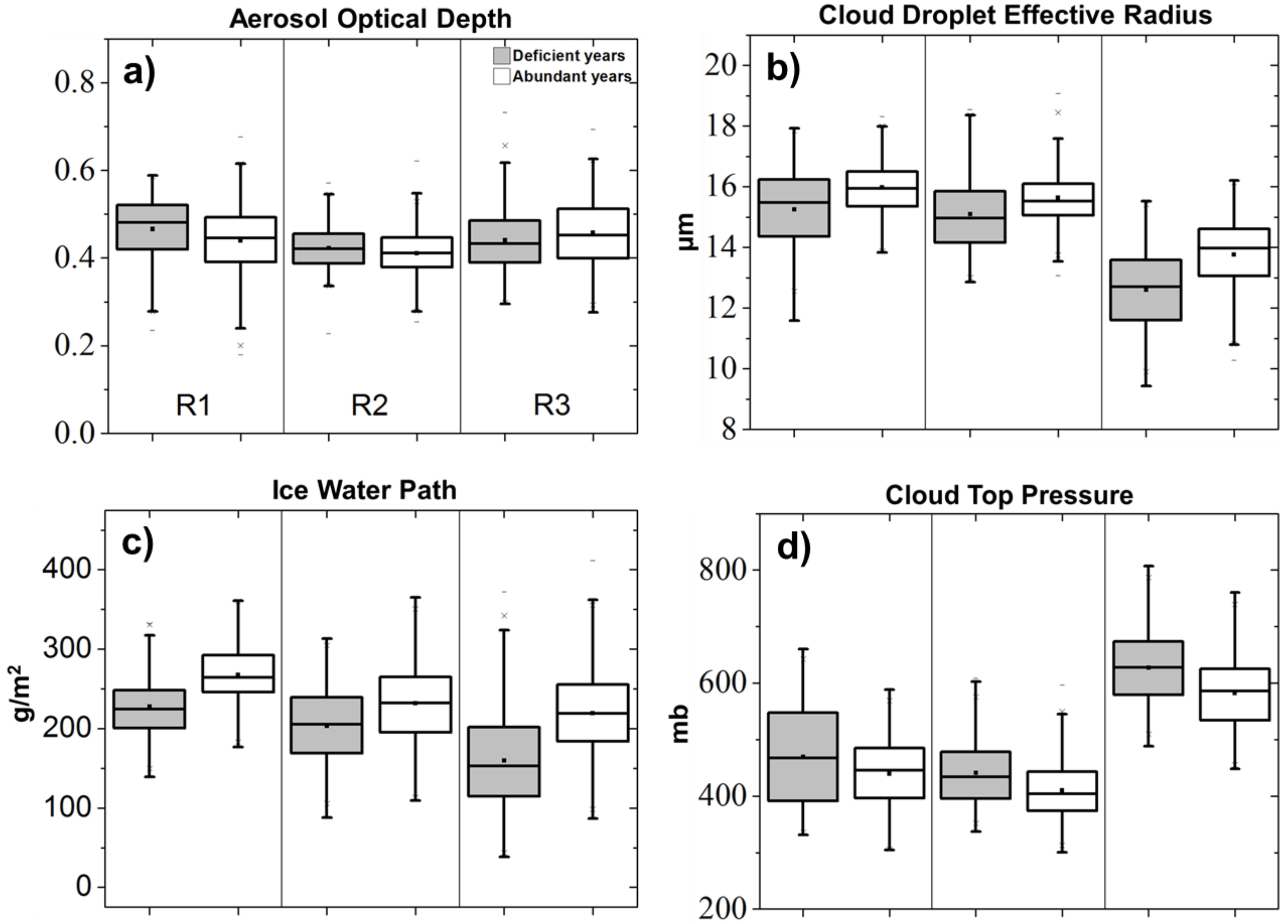

Figure S3 | Box and Whisker plot of region-specific JJAS absolute values at the pixel level during deficient (grey) and abundant (white) rainfall years. (a) AOD, **(b)** CDER, **(c)** IWP and **(d)** CTP. Mean values during deficient and abundant rainfall years are significantly different at $P < 0.10$.

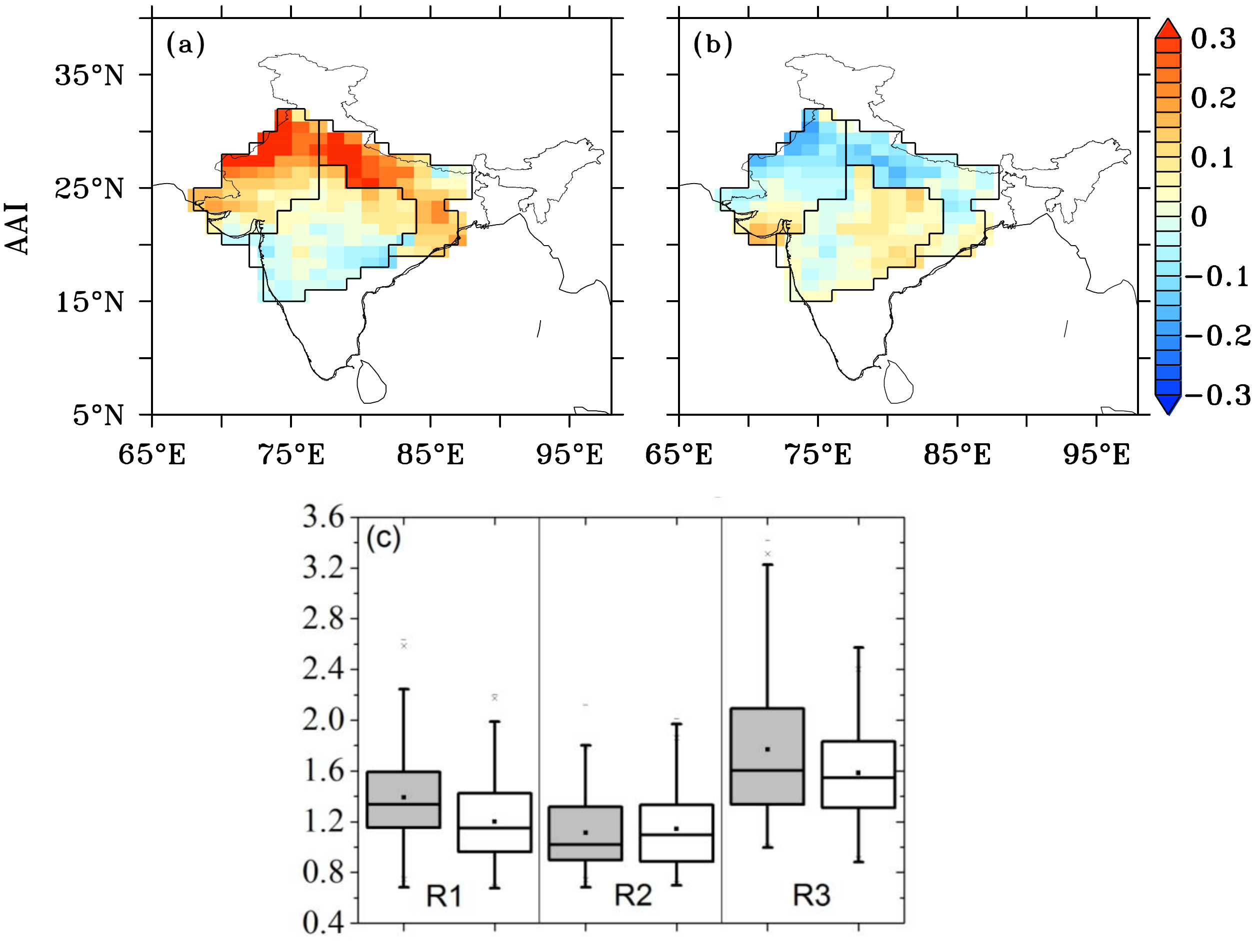

Figure S4 | Region-specific aerosol absorbing index JJAS anomalies during (a) deficient and (b) abundant rainfall years; and (c) Box and Whisker plot of the absolute values at each pixel. Figure was created using FERRET v7.0 (http://www.ferret.noaa.gov/Ferret/).

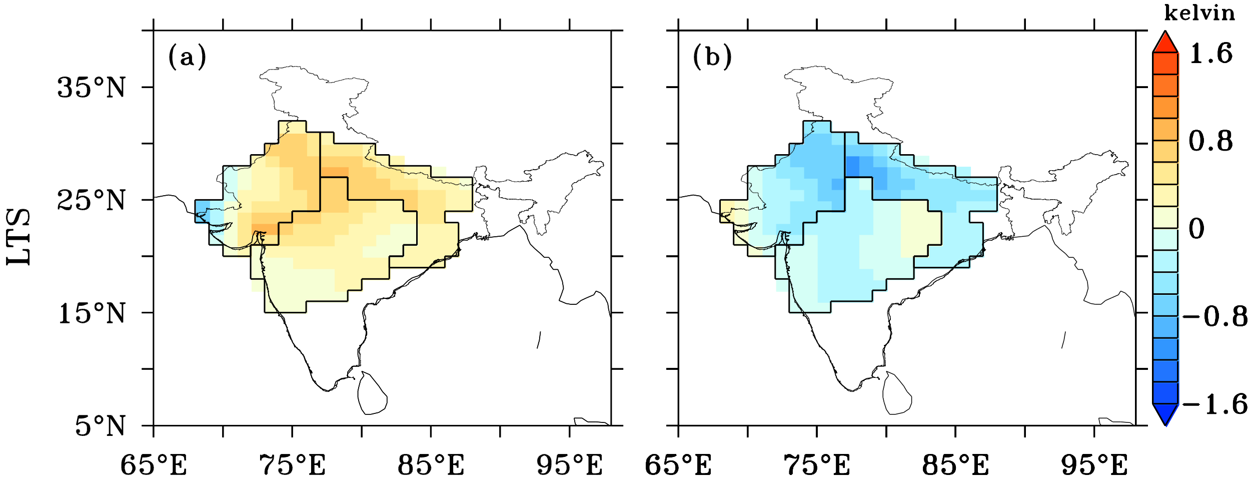

Figure S5 | Region-specific lower tropospheric stability (LTS) JJAS anomalies during (a) deficient and (b) abundant rainfall years. Figure was created using FERRET v7.0 (http://www.ferret.noaa.gov/Ferret/).

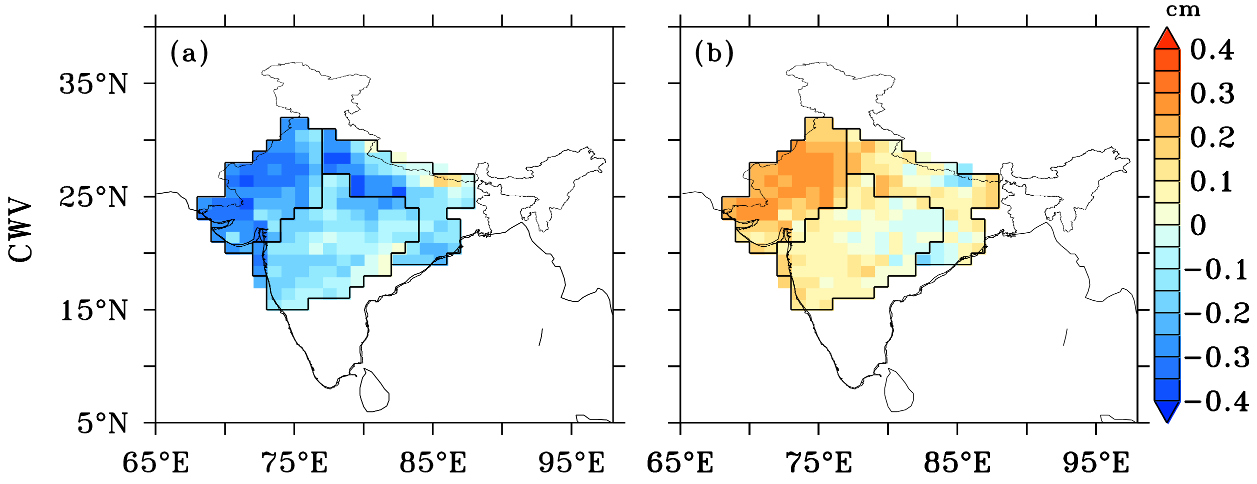

Figure S6 | Region-specific column water vapour JJAS anomalies during (a) deficient and (b) abundant rainfall years. Figure was created using FERRET v7.0 (http://www.ferret.noaa.gov/Ferret/).

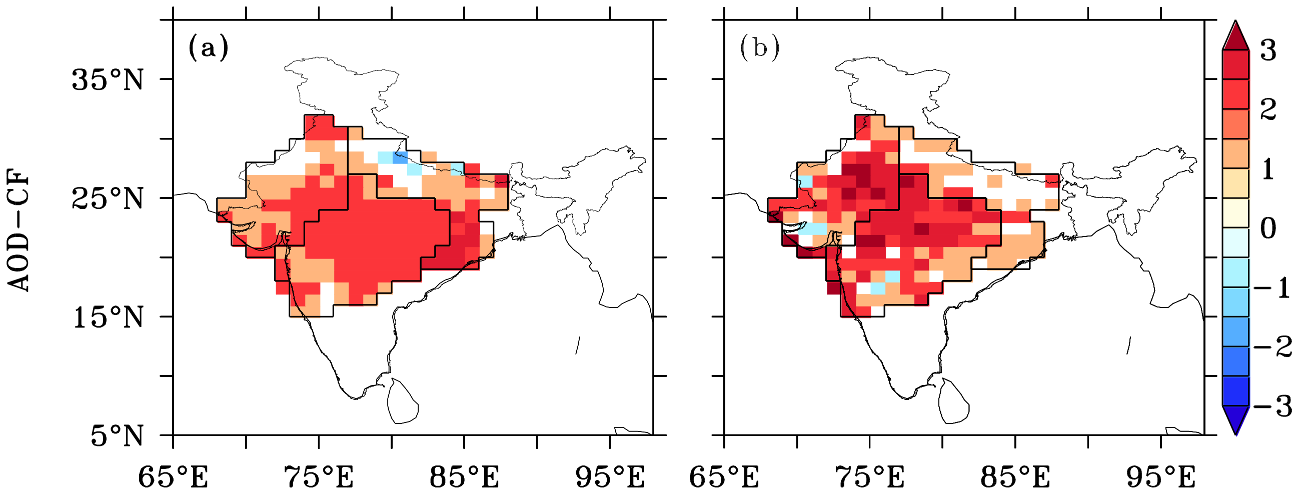

Figure S7 | Temporal correlation cumulative frequency of AOD and CF during (a) deficient and (b) abundant rainfall years (significant at P < 0.10). Figure was created using R statistical tool v3.3.1 (https://www.r-project.org/) and FERRET v7.0 (http://www.ferret.noaa.gov/Ferret/).

Table S1. Number of break periods and days per break period during abundant and deficient rainfall years, based on the Indian Meteorological Department rainfall observations.

Year (# breaks : # days per break)		
Regions	Deficient rainfall years	Abundant rainfall years
R1	2002 (2 : 3, 5); 2004 (3 : 4, 3 ,7); 2009 (3 : 7, 3, 3)	2007 (1 : 3)
R2	2000 (3 : 5, 3, 6); 2002 (2 : 11, 3); 2009 (3 : 4, 3, 7)	2005 (2 : 4, 3); 2006 (1 : 11); 2007 (1 : 3)
R3	2000 (1 : 4); 2009 (1 : 4)	No breaks
